# Estradiol-dependent hypocretinergic/orexinergic behaviors throughout the estrous cycle

**DOI:** 10.1007/s00213-022-06296-1

**Published:** 2022-12-26

**Authors:** Hye Ji J. Kim, Samuel A. Dickie, Robert B. Laprairie

**Affiliations:** 1 College of Pharmacy and Nutrition, Health Sciences Bldg. Rm 3B36, University of Saskatchewan, 107 Wiggins Rd, Saskatoon, SK S7N 5E5 Canada; 2 Department of Pharmacology, College of Medicine, Dalhousie University, Sir Charles Tupper Medical Bldg. Rm 6E, 5850 College Dr, Halifax, NS B3H 4R2 Canada

**Keywords:** Orexin system, Orexin receptor antagonists, Estrous cycle, Estradiol, Progesterone, HPG axis, Arousal, Feeding, Reward, Anxiety

## Abstract

**Rationale:**

The female menstrual or estrous cycle and its associated fluctuations in circulating estradiol (E2), progesterone, and other gonadal hormones alter orexin or hypocretin peptide production and receptor activity. Depending on the estrous cycle phase, the transcription of prepro-orexin mRNA, post-translational modification of orexin peptide, and abundance of orexin receptors change in a brain region-specific manner. The most dramatic changes occur in the hypothalamus, which is considered the starting point of the hypothalamic-pituitary–gonadal axis as well as the hub of orexin-producing neurons. Thus, hypothalamus-regulated behaviors, including arousal, feeding, reward processing, and the stress response depend on coordinated efforts between E2, progesterone, and the orexin system. Given the rise of orexin therapeutics for various neuropsychiatric conditions including insomnia and affective disorders, it is important to delineate the behavioral outcomes of this drug class in both sexes, as well as within different time points of the female reproductive cycle.

**Objectives:**

Summarize how the menstrual or estrous cycle affects orexin system functionality in animal models in order to predict how orexin pharmacotherapies exert varying degrees of behavioral effects across the dynamic hormonal milieu.

## Introduction

The hypothalamus-pituitary–gonadal (HPG) axis drives the female reproductive cycle, termed the menstrual cycle in humans and non-human primates or the estrous cycle in preclinical models such as mice, rats, and pigs. Characterized by rhythmic fluctuations in circulating gonadal hormones such as estrogens (namely, estradiol [E2]) and progesterone, the estrous cycle influences the actions of numerous neuropsychiatric drug classes, including orexin or hypocretin receptor antagonists (Brot et al. 1995; Díaz-Véliz et al. 2000; Kinkead et al. 2000; Silveyra et al. 2007a; Benmansour et al. 2009; Yohn et al. 2020). Preceding their clinical approval for use in sleep disorders such as insomnia (reviewed in Muehlan et al. 2020), both selective orexin receptor antagonists (SORAs) and dual orexin receptor antagonists (DORAs) were and continue to be tested in animal demonstrations of abnormal feeding like binge eating (Haynes et al. 2002; Piccoli et al. 2012), substance-use disorder (reviewed in Perrey and Zhang 2020), and stress-induced anxiety-like behaviors (Vanderhaven et al. 2015; Salvadore et al. 2020). The orexin system functionally interacts with the HPG axis (reviewed in Silveyra et al. 2010) such that orexin-mediated behaviors are modulated in part by estrous cycle phases (Deurveilher et al. 2008; Arthaud et al. 2022; Funabashi et al. 2009; Zhou et al. 2012; Amodeo et al. 2018).

This review article aims to first summarize how estrous cycle phases influence the brain region-specific expression of orexin receptor mRNA, as well as the abundance of orexin peptides and G protein-coupled receptors (GPCRs) in mice, rats, and pigs. Next, this review will explore how orexin-mediated arousal, feeding, reward processing, and anxiety-like behaviors are changed throughout the estrous cycle. These behavioral topics were chosen based on the availability of original data articles with evidence of both the estrous cycle and orexin system built in. The second part of the review will also identify gaps in the preclinical literature, which upon being addressed, may increase the clinical translatability of these neuroendocrine-orexin interactions from preclinical models into humans. Despite the handful of studies reporting that post-menopausal women have lower plasma concentrations of orexin A (OXA; El-Sedeek et al. 2010; Messina et al. 2013), there are no human data connecting active menstrual cycle phases with the endogenous orexin system. There is also no clinical literature on the pharmacodynamic effects of exogenous orexin receptor antagonists across the human menstrual cycle. For these reasons, this review will focus on the available preclinical data in mice, rats, pigs, and nonhuman primates.

This research topic represents a small but growing area of interest, as (1) pharmacotherapeutics targeting the orexin system are gaining clinical traction and (2) preclinical studies are becoming increasingly cognizant of sex and female reproductive cycle influences on drug efficacy and safety. Knowledge synthesis in this relatively young field requires the capture of a broad swath of preclinical studies to gain the best perspective. By providing the most up-to-date preclinical evidence on this research topic, the aim of this review article is to identify shortcomings within the literature in order to promote sex- and neuroendocrine-specific therapeutic application of emerging orexin receptor antagonists.

## The menstrual or estrous cycle as a function of the HPG axis

The HPG axis controls sexual reproduction through a series of hormones that are triggered by the hypothalamic production of gonadotropin-releasing hormone (GnRH) (reviewed in Plant 2015). Upon release of GnRH, the pituitary secretes LH and FSH, leading to the synthesis and circulation of gonadal hormones such as testosterone from the testes and estrogens and progesterone from the ovaries (Richards et al. 1976; Baram and Koch 1977; Bruni et al. 1977). These gonadal hormones encourage reproductive processes, including sexual maturation, the menstrual or estrous cycle, fertilization, and pregnancy (reviewed in Plant 2015).

The human menstrual cycle occurs over 28 days, while other species like non-human primates and pigs undergo 20–30- and 21-day female reproductive cycles, respectfully (Hunnell et al. 2007; Park et al. 2022). Much of the estrous cycle data included in the current review will focus on pigs in addition to mice and rats. The mouse and rat estrous cycles span 4–5 days and consist of phases: proestrus, estrus, metestrus, and diestrus (Byers et al. 2012; Ajayi and Akhigbe 2020). Early proestrus is characterized by a gradual elevation in plasma E2 concentration along with a lull in circulating progesterone, which stimulates ovulation in the follicular phase (Byers et al. 2012; Ajayi and Akhigbe 2020). This is followed by sudden rises in progesterone, LH, and FSH during middle to late proestrus (Byers et al. 2012; Ajayi and Akhigbe 2020). By late estrus, both E2 and progesterone drop to low levels, which remain decreased until late diestrus (Byers et al. 2012; Ajayi and Akhigbe 2020). After a reduction in concentration during proestrus, estrus, and metestrus, progesterone increases during diestrus to maintain the luteal phase (Byers et al. 2012; Ajayi and Akhigbe 2020). These fluctuations in circulating hormones influence neurological function as gonadal hormone molecules are lipophilic and readily pass through the blood–brain barrier (reviewed in Diotel et al. 2018). Aside from their syntheses in the ovaries and testes, the brain produces its own supply of sex hormones that partake in HPG axis feedback (reviewed in Brann et al. 2021). In order to overcome the hormonal fluctuations of the estrous cycle in animal studies, gonadectomies — particularly ovariectomies in female animals — are regularly used to limit the effects these gonadal hormones have on preclinical models’ physiology (Wise and Ratner 1980; Giles et al. 2010).

**Fig. 1:**
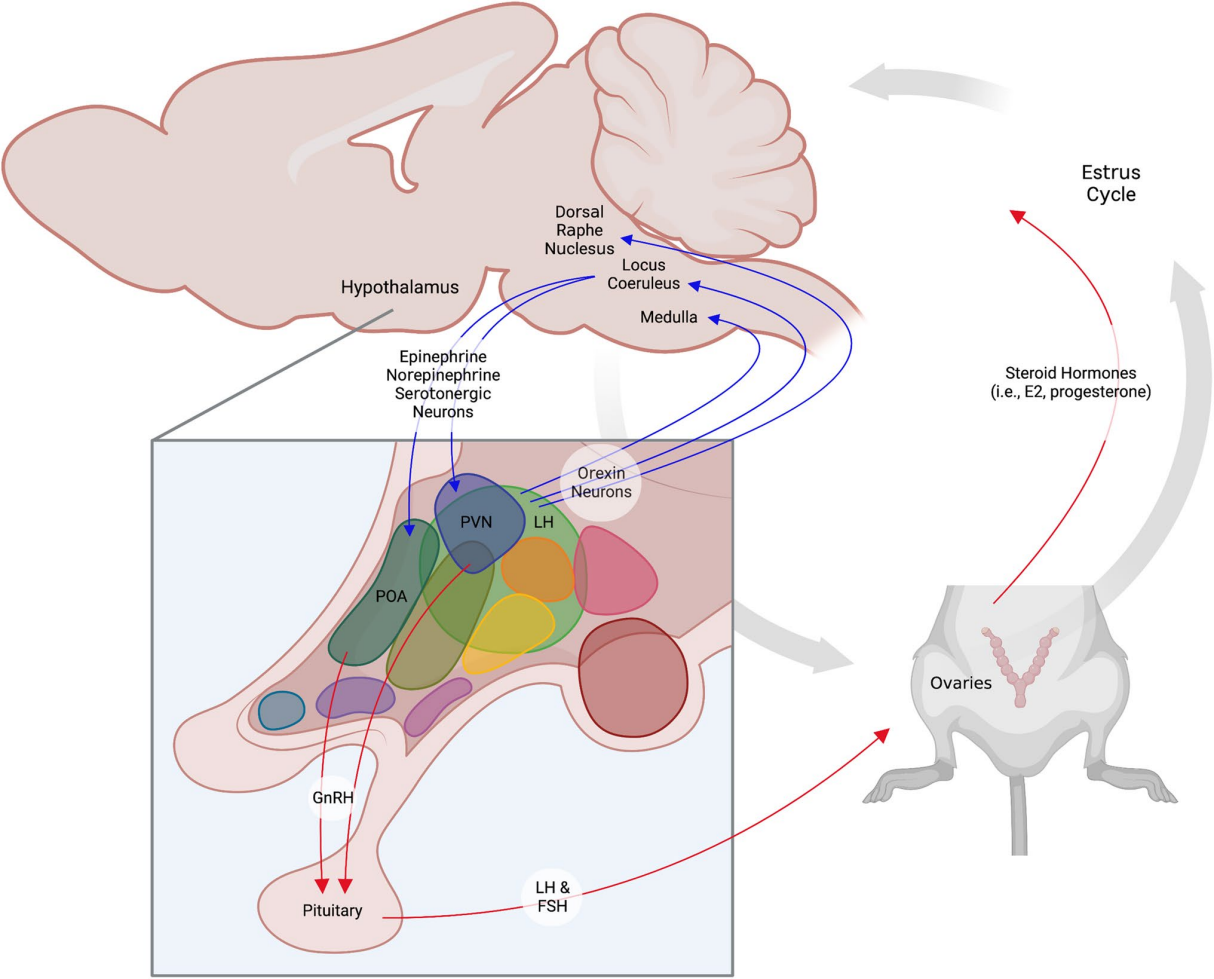
Interactions between the orexin system, HPG axis, and estrous cycle. Orexin neurons from the lateral hypothalamus (LH) project to brain regions innervating the GnRH-producing preoptic (POA) and paraventricular (PVN) areas of the hypothalamus. Neuronal projections are depicted by blue arrows. GnRH release stimulates the pituitary’s production of LH and FSH, which activate the female gonads or ovaries to secrete estradiol (E2), progesterone, and other sex hormones that drive the estrous cycle in female mice and rats. Hormone secretion is represented by red arrows. The figure was created using Biorender.com

The estrous cycle is partly modulated by the orexin system (Silveyra et al. 2007a) (Fig. 1). Orexin-containing neurons project from the lateral hypothalamus to the medulla, dorsal raphe nucleus, and locus coeruleus (Peyron et al. 1998; Date et al. 1999; Peyron and Kilduff 2017), which control epinephrine, norepinephrine, and serotonergic innervations to the paraventricular and preoptic hypothalamus (Pantić 1995, reviewed in Spergel 2019). GnRH release from these hypothalamic regions then triggers gonadal hormone fluctuations, as shown in Fig. 1 (Pantić 1995; reviewed in Spergel 2019). This physiological pipeline reveals the influence that hypothalamic orexins have on circulating E2 and progesterone (Fig. 1). Blocking OX1R and OX2R using the OX1R-selective SORA SB-334867 and the OX2R-selective SORA JNJ-1037049 inhibits GnRH secretion and ovulation in cycling female rats, respectfully (Silveyra et al. 2007a). Conversely, the GnRH receptor antagonist Cetrorelix prevents the otherwise natural pre-ovulatory increases in orexin receptor expression (Silveyra et al. 2007a, b). Just as orexin receptor antagonists disrupt the estrous cycle of rats, direct inhibition of gonadal hormone release blunts hypothalamic orexinergic activity (Silveyra et al. 2007a, b).

## Primer on the orexin system

The orexin or hypocretin system controls a broad range of physiological and psychological functions including arousal or wakefulness (España et al. 2002; reviewed in Alexandre et al. 2013); feeding (reviewed in Sakurai 1999; Muthmainah et al. 2021); reward (reviewed in Plaza-Zabala et al. 2012); energy homeostasis (reviewed in Tsuneki et al. 2012); the multi-modal stress response precipitating anxiety (reviewed in Grafe and Bhatnagar 2018); and reproduction (reviewed in Silveyra et al. 2010). Neuropeptides OXA and orexin B (OXB) are synthesized from the proteolytic cleavage of prepro-orexin (PPO) within the lateral hypothalamus, perifornical area, and posterior hypothalamus (de Lecea et al. 1998; Sakurai et al. 1998). There are approximately 3,000 orexin neurons in the rat hypothalamus, while the human hypothalamus contains up to 70,000 (de Lecea et al. 1998; Sakurai et al. 1998; Soya and Sakurai 2020). Endogenous orexins bind to GPCRs, orexin receptor type 1 (OX1R), and orexin receptor type 2 (OX2R), with varying affinities. OXA has a higher binding affinity to OX1R as compared to OX2R, while OXB almost exclusively stimulates the latter (de Lecea et al. 1998; Sakurai et al. 1998; Yin et al. 2016). Orexin receptors are highly conserved among mammals, as human and rat OX1R and OX2R share 94 and 95% expression homology, respectively (de Lecea et al. 1998; Sakurai et al. 1998; Trivedi et al. 1998; Marcus et al. 2001; Wang et al. 2018). Orexin receptor localization throughout brain regions is also very similar between mice and rats (Marcus et al. 2001; Ch’ng and Lawrence 2015; Chen et al. 2006; Ikeno and Yan 2018). The activation of orexin receptors triggers intracellular cascades, most often resulting in Ca^2+^-dependent membrane depolarization and excitatory neurotransmission (Uramura et al. 2001; Xia et al. 2009; Nakamura et al. 2010; Woldan-Tambor et al. 2011). Rat brain regions that express both OX1R and OX2R include the dentate gyrus, septum, amygdala, thalamus, hypothalamus, periaqueductal gray, dorsal raphe nucleus, ventral tegmental area, and nucleus tractus solitarii (Marcus et al. 2001). Within the hypothalamus, orexin signaling occurs alongside other neurotransmitter and neuroendocrine systems to modulate the HPG axis (Pantić 1995; Laflamme et al. 1998; Horvath et al. 1999; Muraki et al. 2004; Liu and Gao 2007).

## Brain region-specific orexin mRNA, ligand peptide, and receptor expression across the estrous cycle

The gene expression and protein quantities of orexin peptides and receptors change throughout the female reproductive cycle. Although this evidence is limited to rat and pig models, it alludes to a bidirectional relationship between the orexin system and the HPG axis. In the thalamus, midbrain, and medulla of ovary-intact female rats, OXA protein concentrations are highest during the afternoon of proestrus when circulating E2 is elevated and progesterone levels are low (Russel et al. 2001). Unfortunately, there is no information on how PPO, OXB, and the orexin receptor subtypes are regulated by the estrous cycle in these brain regions. When the hypothalamus is dissected as one entity, its protein levels of OXA during late proestrus are reported to be decreased by some sources (Russel et al. 2001), while shown to be increased in other studies (Porkka-Heiskanen et al. 2004). This discrepancy may be due to varying criteria for “afternoon of proestrus” as well as the nonspecific manner of dissecting the whole hypothalamus. Porkka-Heiskanen and colleagues (2004) also found that OXB protein and OX1R mRNA expression are greater in the hypothalamus of late proestrus rats occupying the early follicular phase. Separating the hypothalamus into its distinct cell populations may provide more insights into these mRNA and protein data.

The mediobasal, anterior, preoptic, and salk median eminence regions of the hypothalamus display different patterns of mRNA expression and protein concentration of PPO, OXA, OXB, OX1R, and OX2R. Each of these hypothalamic areas represent unique functions related to homeostasis, whereby the mediobasal hypothalamus (MBH) specifically contains the arcuate nucleus, which releases GnRH to activate the HPG axis (reviewed in Korf and Møller 2021). In ovary-intact female pigs, the MBH presents with the lowest expression of PPO mRNA during estrus or the late follicular phase, marked by increased and decreased levels of E2 and progesterone, respectively (Maleszka et al. 2013). MBH protein concentrations of OXA and OXB escalate in the subsequent luteal phase of pigs (Maleszka et al. 2013). The trend differences between PPO and OXA/OXB may reflect a feedback mechanism which, in part, relies on hypothalamic and pituitary GnRH secretion. In both the mediobasal and anterior regions of the rat hypothalamus, PPO protein is most abundant during the afternoon of proestrus (Silveyra et al. 2007b). Despite the lateral hypothalamus being the main site of PPO synthesis and cleavage into OXA and OXB, other hypothalamic regions show evidence of orexin peptide processing to a lesser extent (de Lecea et al. 1998; Sakurai et al. 1998). Elevated PPO concentrations during proestrus signify an impending surge of its cleavage products in the later follicular phase. Within the preoptic area of the pig hypothalamus, OXA protein levels rise during estrus and are maintained through the early luteal phase, while OXB protein quantity increases later on (Maleszka et al. 2013). This pattern suggests contrasting roles between OXA and OXB in the preoptic area — a region largely responsible for thermoregulation contributing to sleep — throughout the estrous cycle (reviewed in Rothhaas and Chung 2021). In the salk median eminence, or the anatomical interface between the hypothalamus and pituitary, PPO mRNA expression increases during estrus, corresponding to OXA and OXB reaching high levels in late proestrus of pigs (Maleszka et al. 2013).

In all regions excluding the MBH, OX1R, and OX2R mRNA and protein concentrations are largest during late proestrus in pigs (Kaminski et al. 2010a, b). In the MBH, only OX2R protein is significantly increased during late proestrus in this same animal model (Kaminski et al. 2010a, b). Receptor mRNA expression gives clues about ligand binding, the magnitude of downstream intracellular events, transcription autoregulation, and/or receptor reorganization on the plasma membrane based on receptor activity. The preoptic and salk median eminence regions display a positive association between circulating E2 levels and orexinergic signaling in pigs (Coryn et al. 1979; Kaminski et al. 2010a, b; Maleszka et al. 2013; Park et al. 2022). In the MBH, however, the opposite trend is observed (Maleszka et al. 2013; Park et al. 2022). Female rats in late proestrus have higher amounts of OX1R and OX2R in their MBH (Silveyra et al. 2007b), which highlights the species-specific nature of estrous cycle-dependent orexin functionality in the MBH.

The pituitary gland’s constituents of the orexin system are also affected by the HPG axis and estrous cycle of pig and rodent models. In the posterior pituitary of pigs, transcriptional data pointing to the abundances of PPO, OXA, OXB, OX1R, and OX2R is greater during estrus — or the late follicular phase, when circulating E2 levels begin to decrease — as well as during metestrus or early luteal phase when E2 settles to its lowest point (Kaminski et al. 2010a, b; reviewed in Kaminski and Smolinska 2012; Smolinska et al. 2014). While the anterior pituitary receives signals from the hypothalamus to synthesize its own hormones, the posterior region serves as storage for hypothalamically produced hormones. With respect to the anterior pituitary, PPO, OXA, and OX1R protein concentrations are higher throughout metestrus and diestrus in pigs (Kaminski et al. 2010a, b; Smolinska et al. 2014). Protein levels of OXB and its more readily bound OX1R are meanwhile lower during this period (Kaminski et al. 2010a, b; Smolinska et al. 2014). In such an area where GnRH is synthesized and released on the basis of low E2 (Adams et al. 2018), OXA activity at OX1R in the anterior hypothalamus is positively and negatively associated with E2 and GnRH release, respectively. These patterns are again different in other species, such as rats, where OX1R and OX2R are most abundant during late proestrus (Silveyra et al. 2007b).

The compilation of rat and pig data summarized here emphasizes brain region specificity regarding estrous cycle-dependent orexinergic activity. Additionally, OXA and OXB signaling are differentially regulated via gene expression and protein abundance across the hormonal milieu. Finally, the degree of dissimilarity between preclinical species presents as a barrier in translation to humans. Future work in characterizing the link between the female reproductive cycle and brain region-specific orexinergic activity should focus on a single species with the highest degree of genetic homology to humans when it comes to the orexin system.

## Orexin-mediated arousal or wakefulness across the estrous cycle

A variety of biological functions are dually regulated by the orexin system and estrous cycle. Wakefulness represents one of these functions that is enhanced by both neuropeptides in preclinical models (Swift et al. 2020; Kim et al. 2021; De Luca et al. 2022) and humans (reviewed in Scammel et al. 2017; Rahman et al. 2019). Proestrus rats with higher and lower circulating E2 and progesterone, respectively, exhibit less paradoxical sleep (Schwierin et al. 1998; Swift et al. 2020) and are the most vigilant during the day (reviewed in Bangasser et al. 2018; Lovick and Zangrossi 2021). To this effect, progesterone is thought to inhibit GnRH-induced wakefulness and promote sleep (Lancel et al. 1996; Camacho-Arroyo et al. 1999; Kim et al. 2018; Sun et al. 2019). Although there is no evidence to connect progesterone and orexin functionality in sleep, there have been attempts to relate the latter to E2. In ovariectomized (OVX) female rats with little E2 functionality, sleep deprivation intensifies c-Fos immunoreactivity in several arousal-promoting limbic and neuroendocrine nuclei while reducing neuronal activity in the sleep-promoting ventrolateral preoptic nucleus (Deurveilher et al. 2008). E2 supplementation to sleep-deprived OVX rats not only elevates c-Fos neuronal activity in the bed nucleus of the stria terminalis and tuberomammillary nucleus but also within the orexin-containing neurons of the hypothalamus (Deurveilher et al. 2008). These results suggest that E2 recruits hypothalamic orexin neurons to mediate wakefulness (Deurveilher et al. 2008), exemplifying a positive association between these two neuromodulatory systems when it comes to sleep promotion. Another study showed that when orexin function is knocked out to produce a narcolepsy type 1 phenotype, females of this mouse strain display normal estrous cycling despite the severity of their catalepsy being phase-dependent (Arthaud et al. 2022). In the absence of orexin activity, longer and more frequent cataplectic episodes occur during estrus when both E2 and progesterone levels have dropped (Arthaud et al. 2022). Interestingly, vigilance, time awake, slow-wave sleep, and paradoxical sleep do not change across the estrous cycle in these knockout mice (Arthaud et al. 2022) despite these sleep–wake characteristics being phase-dependent in wild-type animals (Schwierin et al. 1998; Swift et al. 2020; Arthaud et al. 2022). Thus, some aspects of arousal and wakefulness such as catalepsy rely on orexin/estrous cycle signaling, but not all (Schwierin et al. 1998; Swift et al. 2020; Arthaud et al. 2022).

Although the investigations by Deurveilher and colleagues (2009) and Arthaud and colleagues (2022) demonstrate a degree of interdependence between the orexinergic activity and E2 abundance with respect to sleep, more research is needed to understand how the inhibition of each orexin receptor subtype produces unique sedation profiles across the female reproductive cycle. Work isolating the effect of orexins on progesterone-mediated wakefulness and sleep also represents a gap in the literature.

## Orexin-mediated feeding across the estrous cycle

The orexin system and estrous cycle both contribute to feeding behavior (reviewed in Sakurai 1999; Asarian and Geary 2006). OXA promotes appetite by delaying the satiety sequence to prolong homeostatic and hedonic feeding in mice and rats (Arafat et al. 2014; Rodgers et al. 2000). Moreover, fasting increases the number of lateral hypothalamic orexin neurons expressing the cellular activation marker, phosphorylated cyclic AMP response element-binding protein (pCREB), in rats (Funabashi et al. 2009; Fukushima et al. 2015). Orexigenic promotion of feeding has also been justified as a secondary or subsequent outcome of arousal (España et al. 2002; reviewed in Burdakov 2004). Similar to orexin, progesterone stimulates appetite and weight gain, but only in the presence of E2 (Wade and Schneider 1992). Although progesterone encourages feeding in OVX mice at higher, non-physiological doses, it is thought to have a lesser role in ingestive behaviors as compared to E2 (reviewed in Butera 2010). Of all the female reproductive cycle phases, preclinical models such as non-human primates, rats, and mice feed less during proestrus and estrus when they embody higher plasma E2 and lower progesterone (Peterson 1976; Wade and Gray 1979; Bielert and Busse 1983; Fantino and Brinnel 1986; Roth et al. 2005; Abdulhay et al. 2014; reviewed in Richard et al. 2017). Conversely, diestrus is seen with greater food consumption by both women and female rodents (Peterson 1976; Wade and Gray 1979; Fantino and Brinnel 1986; Abdulhay et al. 2014; reviewed in Hirschberg 2012; Richard et al. 2017). Finally, OVX female rats eat more than their cycling counterparts (Wade 1975; Blaustein and Wade 1976; Asarian and Geary 2002), which can be reversed by E2 supplementation (Wade 1975; Asarian and Geary 2002).

As it relates to the orexigenic system, E2-treated OVX mice have decreased hypothalamic expression of appetite-inducing genes for agouti-related peptide, neuropeptide Y, prepromelanin-concentrating hormone, and the orexin peptides (Santollo et al. 2012). There are no studies distinctly relating progesterone and orexinergic activities. Nonetheless, when normally cycling female rats are subjected to fasting before glucose injections, those in late diestrus — with higher and lower levels of circulating E2 and progesterone, respectively — are more sensitive to post-fasting glucose administration (Funabashi et al. 2009). Late-diestrus rats also present with a larger glucose-induced decrease in pCREB-expressing orexin neurons in their lateral hypothalamus (Funabashi et al. 2009; Fukushima et al. 2015). This indicates that high E2 and low progesterone conditions have a greater influence on orexigenic signaling.

The studies by Fukushima and colleagues (2015) suggest that an inverse relationship exists between E2 and orexin activity with regards to feeding behavior throughout the estrous cycle (Funabashi et al. 2009; Fukushima et al. 2015). Given that DORAs influence ghrelin-induced feeding (So et al. 2018) and endogenous orexin activity fluctuates throughout the menstrual or estrous cycle, future studies should utilize different SORAs and clinically relevant DORAs to determine whether their pharmacodynamic effects toward each homeostatic and hedonic food consumption changes across the hormonal milieu.

## Orexin-mediated reward-related behavior across the estrous cycle

Reward or pleasure is a highly sexually dimorphic behavioral process that involves both orexin and gonadal hormone activity. Substance use disorders such as cocaine misuse are more prevalent in female animals (reviewed in McHugh et al. 2018; Radke et al. 2021) and humans (reviewed in Becker et al. 2012; Becker 2016). This is believed to be facilitated by female levels of E2 (Hu et al. 2004; Quinones-Jenab and Jenab 2010; Peart et al. 2022). For example, E2-treated OVX female rats display cocaine self-administration sooner and at lower doses compared to untreated OVX females and males (Hu et al. 2004). When it comes to progesterone’s role in preclinically modeled psychostimulant use disorder, it may have reward-diminishing effects (reviewed in Peltier and Sofuglu 2018). Exogenous administration of progesterone to both OVX and non-OVX female rats leads to reduced reinstatement of cocaine-seeking behavior (Anker et al. 2007). Furthermore, ovary-intact female rats that receive progesterone supplementation display less impulsive choice for cocaine as compared to males (Smethells et al. 2016). As a general rule, normally cycling female rats exhibit the greatest motivation to acquire cocaine during late estrus — when E2 circulating levels are high and progesterone concentrations are low — as compared to all other phases (Roberts et al. 1989; Carroll et al. 2002). In humans, the follicular phase of the menstrual cycle precipitates larger reinforcing effects of cocaine and d-amphetamine (Evans et al. 2002; White et al. 2002; reviewed in Joyce et al. 2021). Orexin signaling is similarly implicated in cocaine use disorder, as behaviors such as locomotor sensitization, self-administration, and seeking depend on OXA-OX1R activity in rats (Borgland et al. 2006; España et al. 2011; Matzeu et al. 2021).

Unfortunately, there is no clear evidence of estrous cycle effects on orexin-mediated cocaine-induced behaviors. In the only study considering all three factors (estrous cycle, orexin system, and cocaine use disorder), the OX1R-selective antagonist SB-334867 was shown to attenuate cocaine-induced hyperlocomotion in both sexes of rats while being detected at higher plasma concentrations during the estrus phase in females (Zhou et al. 2012). The latter point alludes that low E2 conditions amplify the absorption and distribution of SB-334867 to target tissues, leading to a higher maximum plasma concentration of the drug. Although the estrous cycle impacts SB-334867 pharmacokinetics, its phases do not dictate changes in cocaine-induced behaviors according to this study (Zhou et al. 2012). Aside from cocaine use disorder, adolescent binge drinking induces higher OX1R and OX2R mRNA expression in the frontal cortex of female rats as compared to male rats (Amodeo et al. 2018). However, there are no intra-female or estrous cycle phase-dependent differences (Amodeo et al. 2018). Altogether, more research is needed to understand how different substance use disorders (i.e., psychostimulant versus CNS depressant misuse) are affected by SORAs and clinically approved DORAs across the female menstrual or estrous cycle.

## Orexin-mediated anxiety-like behavior and panic across the estrous cycle

Studies exploring the link between orexin and anxiety-like behaviors throughout the estrous cycle have largely focused on hormonal changes, ovariectomy, and stress models rather than pharmacological manipulation of the orexin system. Stressful stimuli are received by the orexin system, activating the HPA axis to release corticosterone in mice and rats (Winsky-Sommerer et al. 2004; Ida et al. 2000). Depending on the duration (acute versus repeated or chronic), as well as the overall severity of the stressor, the orexin system is believed to shape stress-coping behaviors (reviewed in Grafe and Bhatnagar 2018). Various modalities of acute stress leading to hyperactivation of the orexin system can precipitate anxious phenotypes both clinically and preclinically (Grafe et al. 2018; Salvadore et al. 2020; Prajapati and Krishnamurthy 2021). E2 is also linked to arousal (Schwierin et al. 1998; Swift et al. 2020; reviewed in Bangasser et al. 2018; Lovick and Zangrossi 2021); where in some instances, excessive E2 causes hypervigilance and panic in cycling women and animal models (reviewed in Soares and Zitek 2008; Glover et al. 2020; Tenorio-Lopes et al. 2020). Unlike E2, exogenous progesterone induces anxiolytic effects that either stand alone or produce synergistic outcomes with E2 in rodents (Frye and Walf 2004; Flores et al. 2020; Sovijit et al. 2021). The relationship between progesterone and orexin in anxiety-like behaviors remains undetermined.

The several datasets describing estrogenic activity in orexin-knockout mice suggest that anxiety is interdependently controlled by these neuromodulatory systems. In an investigation by Easton et al. (2006), OVX mice were intracerebrally injected with orexin conjugated to the neurotoxin saporin before being treated with E2. Compared to orexin-ablated OVX mice deprived of hormonal replacement, those administered E2 exemplify decreased sensory responsiveness and less fearfulness (Easton et al. 2006). These results demonstrate that E2 normalizes anxiety-like behaviors in the absence of orexin activity (Easton et al. 2006). In another more recent study, neonatal maternal separation (NMS) was used to model panic in the form of CO_2_-induced hyperventilation (Tenorio-Lopes et al. 2020). NMS rats with increased hypothalamic concentrations of OXA display greater symptom severity, while OVX in these rats normalizes these panic responses (Tenorio-Lopes et al. 2020). Moreover, both ovary-intact and proestrus mice embody greater E2 abundance and demonstrate progressively higher ventilation responses (Tenorio-Lopes et al. 2020). In the first ever orexin/estrous cycle experimental design using an OX1R-selective SORA, SB-334867 was shown to prevent panic in NMS-subjected proestrus rats, signifying that phase-modulated hyperventilation relies on basal orexin tone (Tenorio-Lopes et al. 2020). These results call attention to the efficacy of SB-334867 to treat hyperventilation-associated panic in cycling females, specifically those occupying the follicular or pre-ovulatory phase. Next steps to this research entails the use of OX2-selective SORAs and clinically relevant DORAs to better distinguish which of the two orexin receptor subtypes is more estrous cycle-dimorphic when it comes to panic-like behaviors. Anxiety induced by different modalities and temporal patterns of stress should also be evaluated.

## Conclusion

Orexinergic signaling has long been known to regulate the HPG axis (reviewed in Silveyra et al. 2010). Orexin activity within the hypothalamus is positively associated with E2 functionality (Silveyra et al. 2007b), just as specific time points of the female reproductive cycle influence orexin peptides and receptor expression (Russell et al. 2001; Wang et al. 2003; Porkka-Heiskanen et al. 2004; Silveyra et al. 2007b). This bi-directional relationship is worth noting in numerous behavioral functions, including arousal, feeding, reward processing, and stress-induced affective states. Given the recent clinical approval of DORAs for sleep disorders like insomnia, future research controlling for the menstrual or estrous cycle should prioritize the application of these drugs to the physiological conditions for falling and staying asleep (i.e., catalepsy, decreased body temperature, antinociception, and anti-locomotion, just to name a few). Among the other suggestions made throughout this review, a larger consideration for progesterone-orexin interactions and clearer distinctions between the roles of OX1R and OX2R may help to overcome the paucity of data on this topic. It would also be useful to know which of the orexin receptor subtypes contributes most to orexin’s interactions with E2 and progesterone in regards to functions beyond what is discussed here (i.e., depression-like behavior and cognition). Finally, assessing the pharmacokinetic profiles of SORAs and DORAs across the female reproductive cycle may inform more about their pharmacodynamic effects. The rising interest in orexin pharmacotherapeutics for various neurological and psychiatric disorders necessitates more research on their hormonal effects, as well as alterations in their pharmacology during times of gonadal hormonal fluctuation.
